# Selected articles from the IEEE International Workshop on Genomic Signal Processing and Statistics (GENSIPS'2011)

**DOI:** 10.1186/1471-2164-13-S6-S1

**Published:** 2012-10-26

**Authors:** Ranadip Pal, Yufei Huang, Yidong Chen

**Affiliations:** 1 Department of Electrical and Computer Engineering, Texas Tech University, Lubbock, 79409, USA; 2 Department of Electrical and Computer Engineering, University of Texas at San Antonio, 78249, USA; 3 Department of Epidemiology & Biostatistics, University of Texas Health Science Center at San Antonio, 78229, USA

## Introduction

The 2011 IEEE International Workshop on Genomic Signal Processing and Statistics (GENSIPS 2011) was organized in San Antonio, Texas from December 2-4th. GENSIPS'11 provided a forum for researchers in the signal processing community, other related computational experts, and biomedical scientists to exchange ideas and discuss signal processing challenges due to the high modality of disparate high-throughput data, high variability of the data acquisition, high dimensionality of data, and high complexity of genomics and proteomics systems. The theme of GENSIPS'11 was cancer and computational biology and GENSIPS featured prominent plenary speakers including Dr. John N. Weinstein from UT MD Anderson Cancer Center, Dr. Stephen Wong from Methodist Hospital at Cornell University, Dr. David Nelson from Baylor College of Medicine and Dr. Chung-I Wu from the Beijing Institute of Genomics.

## Articles

This supplement contains extended versions of selected articles from GENSIPS conference proceedings. Each submitted article to the conference was reviewed by a minimum of two reviewers and the top twenty favorably reviewed papers from sixty selected papers (33%) were invited to submit the extended versions for this supplement. The extended journal versions were further reviewed according to rigorous peer-review criteria.

The accepted articles can be broadly categorized into four groups:

### Gene network inference and cancer therapy design

Models of genetic regulatory networks can belong to the class of deterministic or stochastic, discrete or continuous, fine-scale or coarse-scale quantitative models. The availability of experimental data, prior biological knowledge and purpose of modeling frequently guide the selection of the quantitative model to represent the biological network. Sridharan *et al. *[1] provides an approach for Boolean modeling and analysis of the oxidative stress response pathway based on prior biological knowledge. Oxidative stress has been implicated in a variety of diseases including, but not limited to aging and age-related diseases such as cancer, cardiovascular disease, chronic inflammation, and neurodegenerative disorders. Lin and Khatri [2] considers the aberrant behavior of signaling pathways during cancer as faults in an electronic circuit and presents a Max-SAT based automatic test pattern generation (ATPG) algorithm for cancer therapy. In Haider and Pal [3], a Boolean Network inference algorithm from limited time series data utilizing prior biological knowledge on connectivity is presented. The algorithm is validated on synthetic data and experimental transcriptomic measurements from Human Mammary Epithelial Cell line. As compared to two existing Boolean network inference approaches, the proposed algorithm performs better in terms of robustness and estimation of state transitions. Wu *et al. *[4] provides a linear regression approach to model transcription regulation following estrogen stimulation in breast cancer cells. The proposed technique is validated using gene expression and ER*α *Chip-seq data from the MCF-7 cell line. Fine-scale modeling of genetic regulatory networks using stochastic master equation models entails a huge computational complexity and Karim *et al. *[5] presents a computationally inexpensive way to generate the steady state distributions for stochastic master equation models. In Wang *et al. *[6], a quantitative mathematical model to predict macrophage activation patterns following myocardial infarction is reported. The model was validated on experimental data from adult C57 mice. A single type of genomic data is typically not suitable to understand regulation of cell behavior. Vicente *et al. *[7] presents an approach to assess the gain in predictive performance by integrating various types of biological information in network inference.

### Genomic data analysis

For analysis of DNA methylation profiles, Zhang *et al. *[8] presents a non-parametric infinite beta mixture model to cluster DNA methylation expression profiles produced by Illumina Infinium Beadchip. For genome-wide association studies (GWAS), Jia and Zhao [9] applies a dense module search algorithm for locating network modules that are jointly associated with a disease. A restricted search approach is applied for reducing the computational complexity and the strategy is demonstrated on CATIE GWAS dataset for schizophrenia. Taslim *et al. *[10] proposes a quantitative approach using mixture models to characterize patterns of promoter regions and predict novel and alternative promoters. Jahid and Ruan [11] proposes a method for biomarker discovery by combining microarray gene expression profiles and protein-protein interaction networks.

### Prediction of drug effectiveness

Targeted cancer therapy is considered to be a cornerstone for personalized medicine. Research problems in this area include understanding and modeling the mechanisms of action of molecularly targeted drugs and design of combination drug therapies. Li *et al. *[12] presents an integrated experimental and theoretical approach to investigate the mechanism of action and identify pharmacodynamic characteristics of targeted agents based on cell-line platforms. Specifically, tumor cell response is analyzed via the use of fluorescent reporters; dynamics of drug efficacy for different dosages are studied using dynamic modeling; and time-varying parameters are estimated using system identification techniques. Lin *et al. *[2] proposes to predict the ffectiveness of targeted drugs and guide drug selection by modeling the cancer pathway as a Boolean circuit. Kim and Yoon [13] presents an adaptive reference update (ARU) algorithm to search for the optimal drug combination by comparing the response of the current combination against that of the reference combination and beneficially updating the drug concentrations. Application to real and synthetic examples shows that the ARU algorithm outperforms existing algorithms in terms of effectiveness, efficiency, and robustness.

### Analysis workflows

Advancement in measurement technologies provides vast quantities of experimental data on various components of the regulome. This requires new approaches for the systematic generation and analysis of the experimental data. For large-scale protein profiling, Sun *et al. *[14] provides an integrative model for Liquid Chromatography coupled Mass Spectrometry, and apply it to the systematic analysis of key factors that impact the number of identified peptides and quantified proteins, protein quantification error, differential expression results, and classification performance. The presence of extraneous variables caused by the sample variability can significantly affect the statistical analysis of high-throughput genetic data. Hsu *et al. *[15] proposes a novel approach using batch effect correction, a sample selection process, and a semi-supervised clustering method for reducing the confounding and suppression effects induced by the extraneous variables. Rodriguez *et al. *[16] presents an analysis workflow that permits researchers to compare DNA methylation profiles across multiple biological or patient groups. Comparisons can be made at particular regions of a chromosome or across the genome as a whole. This workflow and its suite of features can assist biomedical scientists in conducting methylation profiling projects and facilitate meaningful biological interpretation of their data. Characterizing copy-number variation is a basic method to profile normal and diseased tissue samples, but challenges remain in accurately interpreting the data from a single genome and comparative measurements from groups of sequenced genomes. Janevski *et al. *[17] studies several variations of copy-number analysis approaches to assess the significance and impact of each methodology choice. Huang *et al. *[18] proposes C2Maps platform as an online bioinformatics resource providing biologists with directional relationships between drugs and genes/proteins in specific disease contexts based on network mining, literature mining, and drug effect annotations. Doderer *et al. *[19] analyzes pathway consolidation approaches and provides a user-friendly web-accessible tool that can enable users to extract functional relations of genes across multiple pathway databases. A case study of performing personal genome analysis on a cloud computing environment is presented in Evani *et al. *[20]. The approach can assist researchers in applying existing cloud computing technologies to analyze enormous amount of data generated by next-generation sequencing technologies.

## Competing interests

The authors declare that they have no competing interests.

## Authors' contributions

All authors served as editors for the supplement, with RP serving as the Lead Editor. All authors helped write this editorial.
